# Diagnosis of Stroke on Neuroimaging of COVID-19 Patients in Coma: A Case Series

**DOI:** 10.7759/cureus.13007

**Published:** 2021-01-30

**Authors:** Rathnamitreyee Vegunta, Radhakrishna Vegunta, Venkata R Rokkam, Gurusaravanan Kutti Sridharan

**Affiliations:** 1 Internal Medicine, Westchester Medical Center, Valhalla, USA; 2 Oncology, University of North Dakota, Fargo, USA; 3 Inpatient Medicine, Banner University Medical Center, University of Arizona, Tucson, USA; 4 Internal Medicine, Banner University Medical Center, University of Arizona, Tucson, USA; 5 Internal Medicine, University of Arizona, Tucson, USA

**Keywords:** embolism, hyper coagulopathy, stroke, covid-19

## Abstract

Patients with severe coronavirus disease 2019 (COVID-19) disease suffer from many thrombotic complications including deep vein thrombosis, pulmonary embolism, myocardial infarction (MI), and stroke. Large vessel strokes have been reported in young patients with COVID-19 disease. We report four cases of stroke diagnosed based on CT scan in critically ill individuals treated in the medical intensive care unit in a health facility in New York. All patients were receiving supportive treatment and mechanical ventilation at the time of diagnosis. All patients had impaired consciousness and were unable to wake up after sedation had worn off, prompting further workup. The pathogenesis of stroke could be secondary to the embolic phenomenon vs. hypercoagulopathy in our patients. Stroke should be considered in all COVID-19 patients who present with altered mental status. Severe COVID-19 patients with risk factors of stroke may benefit from therapeutic anticoagulation.

## Introduction

Coronavirus disease 2019 (COVID-19) infection can lead to many complications including deep vein thrombosis, pulmonary embolism, myocardial infarction (MI), and stroke. The exact pathogenesis of these thrombotic complications is not known. We report four cases of stroke presenting as coma who were treated in a health facility in New York over a three-week period between March 30, 2020, through April 17, 2020, while they were undergoing treatment in the medical intensive care unit. All four patients in this series had a positive nasopharyngeal swab for severe acute respiratory syndrome coronavirus 2 (SARS-CoV-2); they had radiographic evidence of viral pneumonia on CT imaging and were diagnosed with severe COVID-19 disease.

## Case presentation

Clinical characteristics, risk factors, laboratory and imaging data, and outcomes of all patients are presented in Table 1.

**Table 1:** Clinical characteristics of four patients presenting with multi-territorial stroke All the lab values mentioned above were collected on the day stroke was diagnosed on imaging *All patients' GCS score was 3/15 after the drugs used for sedation had passed elimination t 1/2 DIC: disseminated intravascular coagulation; IUFD: intrauterine fetal death; COVID-19: coronavirus disease 2019; SOB: shortness of breath; GCS: Glasgow Coma Scale; NIHSS: National Institutes of Health Stroke Scale; CT: computed tomography; CTA: CT angiography; PCA: posterior cerebral artery; ICA: internal carotid artery; CCA: common carotid artery; MRA: magnetic resonance angiography; INR: international normalized ratio; PTT: partial thromboplastin time; WBC: white blood cells; LDH: lactate dehydrogenase; BNP: B-type natriuretic peptide; APLA: antiphospholipid antibody; CRP: C-reactive protein; CVVHD: continuous venovenous hemodialysis; AKI: acute kidney injury; RRT: renal replacement therapy; DNR: do not resuscitate; MI: myocardial infarction; TTE: transthoracic echocardiogram; EF: ejection fraction; PFO: patent foramen ovale; SVT: supraventricular tachycardia; SQ: subcutaneous

Variable	Patient 1	Patient 2	Patient 3	Patient 4
Age (years)	41	67	71	67
Sex	Female	Male	Female	Female
Medical history and risk factors for stroke	22 weeks gestation, DIC/IUFD	Type 2 diabetes mellitus, hyperlipidemia, atrial flutter	Osteoarthritis of hip	Type 2 diabetes mellitus, hyperlipidemia
Home medications	None	Home medications unavailable	None	Amlodipine, metoprolol, atorvastatin, glimepiride, sitagliptin
COVID-19 symptoms	Cough, fever for 7 days	Cough, fever, diarrhea, SOB for 10 days	SOB, fever for 10 days	Fever, cough for 7 days, SOB for 2 days
Admission-stroke onset (days)	4	18	18	6
Signs and symptoms of stroke	Altered mental status, inability to wake up off sedation for 36 hours	Altered mental status, inability to wake up off sedation for 42 hours	Altered mental status, inability to wake up off sedation for 36 hours	Altered mental status, inability to wake up off sedation for 48 hours
GCS score after sedation t 1/2 elimination*	3/15	3/15	3/15	3/15
NIHSS score	Not available; patient intubated and sedated	Not available; patient intubated and sedated	Not available; patient intubated and sedated	Not available; patient intubated and sedated
Vascular territory (CT head)	Right and left middle cerebral artery	Bilateral cerebral hemispheres and left inferior cerebellum. Superimposed foci of intraparenchymal and/or extra-axial acute hemorrhages within the right frontoparietal regions	Left posterior cerebral artery infarct and small infarct in the left splenium of the corpus callosum	Multifocal infarcts in supratentorial and infratentorial compartments and anterior cerebral artery
CTA	No large vessel occlusion	No large vessel occlusion	Occlusion of proximal left PCA. Moderate-severe stenosis of P2 right PCA	Not done
Carotid Doppler	4-5 mm right ICA aneurysm	No stenosis/thrombus at CCA bifurcation	No stenosis/thrombus at CCA bifurcation	Not done
MRA	Not done	Not done	Not done	Not done
INR	1.07	1.13	1.1	1.1
PTT, seconds (25-32)	24.5	28.6	29.8	21
D-dimer, mg/L (<0.59)	>35	>35	20.8	>35
WBC, k/mm^3 ^(4.8-10.8)	37.5	19.5	6.4	24.2
Neutrophils, % (32-70)	93	92.1	60	86
Lymphocytes, % (21-55)	2	1.4	16.6	5
Platelets, k/mm^3^ (160-410)	71	52	274	336
LDH, U/L (125-220)	2,360	1,292	281	618
Haptoglobin (13-281)	<8	Not done	Not done	Not done
Schistocytes	Occasional	None	None	Occasional
Troponin, ng/ml (0.00-0.02)	<0.02	0.02	0.03	0.05
BNP, pg/ml (<100)	467	289	44	67
APLA workup, protein C, S	Negative	Negative	Negative	Negative
CRP, mg/dl (0.00-0.50)	1.2	4.6	2.8	7.0
Ferritin, ug/L (18-370)	1,429	2,627	5,454	3,583
Procalcitonin, ng/ml (0.15-2)	7	1.01	0.07	2.5
Renal failure	Yes	Yes	Yes	Yes
Hemodialysis	CVVHD transitioned to HD and not requiring HD at discharge	HD during the hospital stay; not requiring HD at discharge	AKI self-resolved; did not require HD	RRT not initiated; was made DNR and started palliative care as per HCP wishes
MI	No	No	No	No
TTE	EF: 60-65%; no PFO/endocarditis/septal effects/thrombus	EF: 50-55%; no PFO/endocarditis/septal effects/thrombus	EF: 60-65%; no PFO/endocarditis/septal effects/thrombus	EF: 45-50%; no PFO/endocarditis/septal effects/thrombus
Arrhythmia	None	SVT, A-fib during the hospital stay. Also had baseline A.flutter	None	None
Treatment for stroke	Aspirin, statin	Aspirin, statin	Aspirin, statin	Aspirin, statin
Treatment for COVID-19	Azithromycin, hydroxychloroquine, tocilizumab, pulse dose steroids, plasma	Tocilizumab, azithromycin, hydroxychloroquine, pulse dose steroids, plasma	Azithromycin, hydroxychloroquine, pulse dose steroids	Azithromycin, hydroxychloroquine, pulse dose steroids
Anticoagulation (uninterrupted)	Heparin drip therapeutic with PTT goal of 50-60	Heparin prophylactic 5,000 u SC Q8H	Heparin prophylactic 5,000 u SC Q8H	Heparin prophylactic 5,000 u SC Q8H
Outcome status	Tracheostomy done, discharged to subacute rehab	Tracheostomy done, discharged to subacute rehab	Tracheostomy done, discharged to long-term acute rehab	Deceased

**Figure 1:**
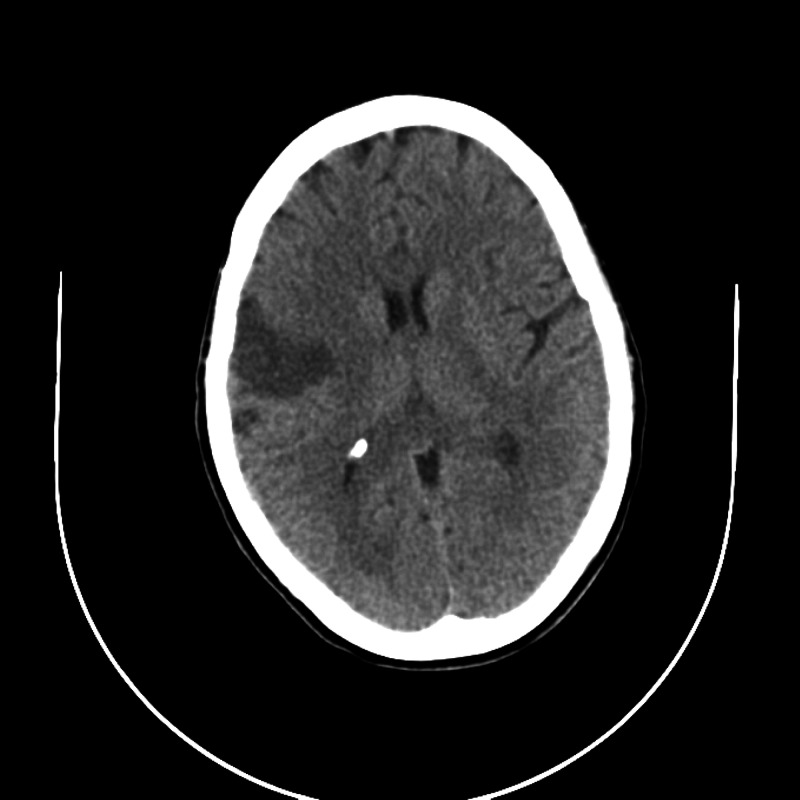
Patient 1 imaging result The image shows acute/subacute small to moderately sized right middle cerebral artery vascular territory infarct and small left middle cerebral artery vascular territory infarct

**Figure 2:**
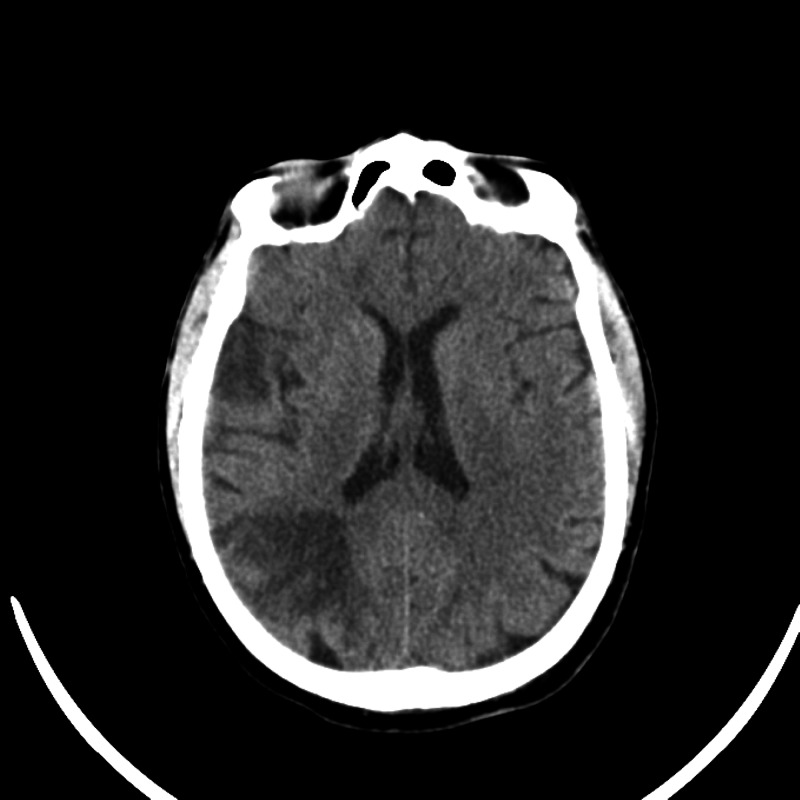
Patient 2 imaging result The image shows multiple likely acute infarcts within the bilateral cerebral hemispheres and left inferior cerebellum

**Figure 3:**
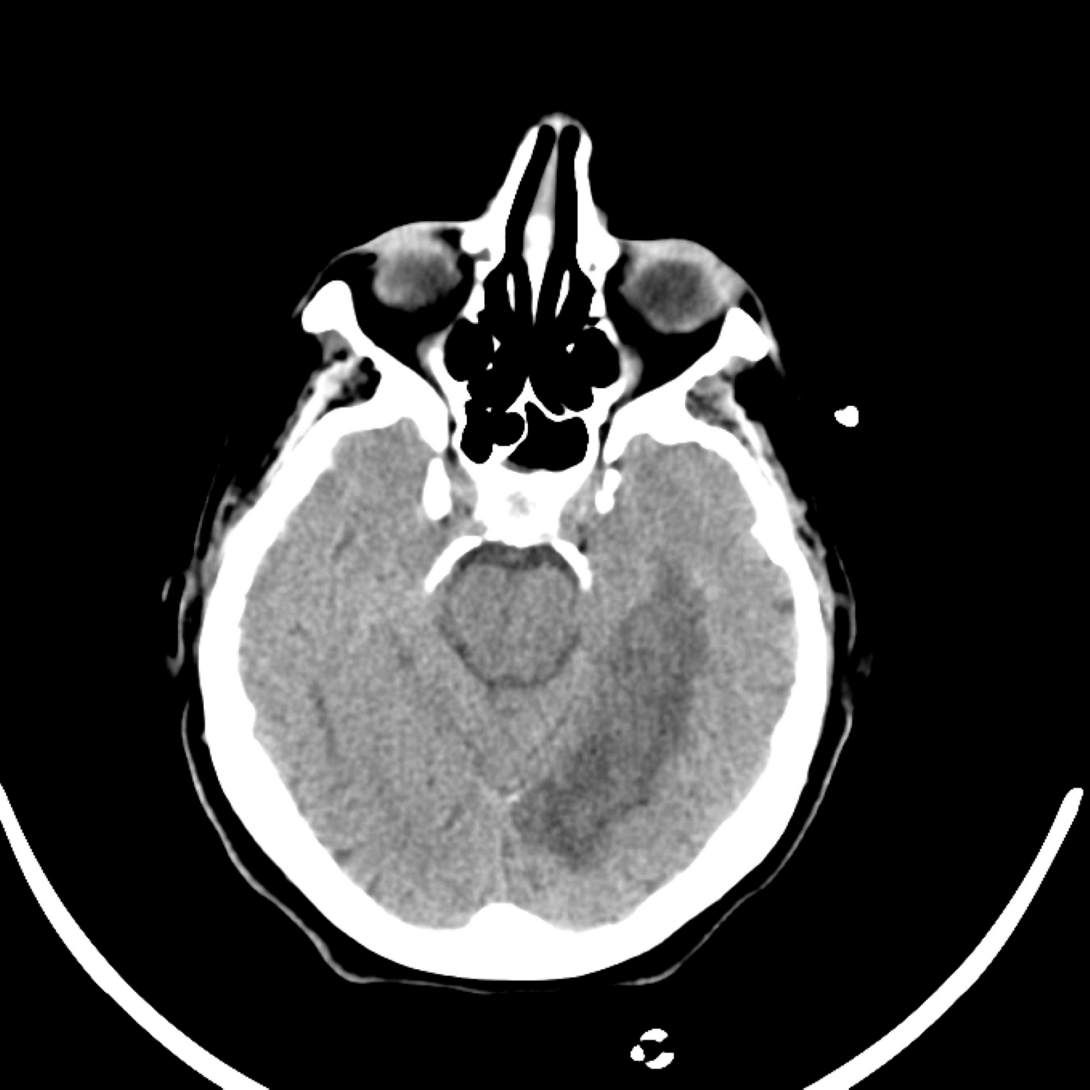
Patient 3 imaging result The image shows acute or subacute left PCA distribution infarct. Additional smaller infarct within the left splenium of the corpus callosum is also seen PCA: posterior cerebral artery

**Figure 4:**
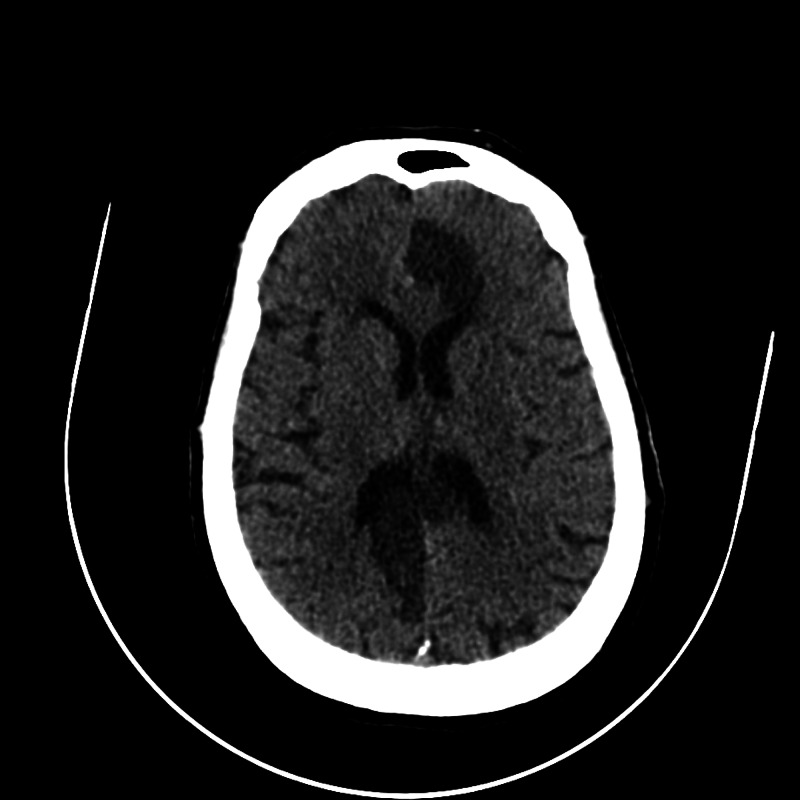
Patient 4 imaging result The image shows multiple small to moderately sized hypodensities within the bilateral supratentorial and infratentorial compartments likely representing multifocal acute-subacute infarcts. Linear hyperdensity within the anterior interhemispheric fissure, which may represent endoluminal thrombus within an anterior cerebral artery, is also seen. Sulcal effacement and mass effect within regions of likely acute-subacute infarcts are observed. Mass effect and partial effacement upon the fourth ventricle can be seen

Imaging results of all patients are illustrated below (Figure 1, Figure 2, Figure 3, Figure 4).

Two of the four patients had known risk factors for stroke. Patient number one was pregnant and was diagnosed with having intrauterine fetal death (IUFD) at 22 weeks gestation and subsequently noted to have disseminated intravascular coagulation (DIC). Patient number two had h/o atrial flutter at admission. He also developed supraventricular tachycardia (SVT) and A-fib during his ICU stay. None of them had patent foramen ovale (PFO)/septal defects/intracardiac thrombus on transthoracic echocardiogram (TTE). All patients had negative blood cultures during their hospital stay. All patients had renal failures at some point during the ICU stay. No patient had a history of transient ischaemic attack (TIA), stroke, or prior coagulopathy. No patients had MI during the ICU stay. Hypercoagulopathy workup done in all the patients was negative for antiphospholipid antibody syndrome and protein C/protein S deficiency.

The symptoms of stroke noted in these patients were altered mental status and the inability to wake up once sedation wore off. All patients were receiving mechanical ventilation at the time of stroke diagnosis. The interval between the onset of symptoms of COVID-19 and the diagnosis of stroke ranged from seven to 27 days. The interval between intubation and diagnosis of stroke ranged from zero to 17 days.

All four patients had an ischemic stroke on CT imaging. Two of those had hemorrhagic conversion (patients 2 and 3), and hence they were not treated with therapeutic anticoagulation after the diagnosis of stroke. Patient 4 had extensive involvement with multiple territories and did not receive therapeutic anticoagulation. Hence, only patient 1 was treated with therapeutic anticoagulation with heparin drip after the diagnosis of stroke. All patients were on prophylactic heparin subcutaneously during their hospital stay (i.e., all patients were found to have stroke despite prophylactic anticoagulation.) None of our patients were treated with tissue plasminogen activator (TPA)/endovascular therapy. Patients 2 and 3 had a hemorrhagic conversion, and patient 4 had extensive multifocal infarcts. Also, the exact onset of stroke was not known in any of our patients.

Even though all our patients had altered mental status, renal failure, and coagulopathy, none of them fit the criteria of thrombotic thrombocytopenic purpura (TTP) as none of them had microangiopathic hemolytic anemia. Hence all the above-mentioned conditions were thought to be secondary to COVID-19 rather than TTP.

All patients received azithromycin, hydroxychloroquine, and high-dose Solu-Medrol® 1 g/day (Pfizer, Brooklyn, NY). Patients 1 and 2 received tocilizumab and plasma in addition to other COVID-19 treatment.

## Discussion

It has been noted that the number of diagnosed cases of stroke in many countries has declined coinciding with the declaration of community spread of novel coronavirus. This is attributed to more people staying at home out of fear of contracting coronavirus [1]. However, several studies have reported a possible association between SARS-CoV-2 infection and an increased risk of stroke [2]. The incidence of thrombotic complications in ICU patients with COVID-19 infections was found to be 31% in a study [3]. Prior studies indicate that a very small proportion (5%) of COVID-19 patients requiring hospitalization are diagnosed with stroke [2-4]. Large vessel thrombotic strokes were reported in young individuals [5]. Some of them do not have classical signs or symptoms of stroke or present with altered mental status and are unable to communicate, making the diagnosis of stroke a challenge. The median time from admission to stroke diagnosis was 12 days in our study compared to 21 days in another study [6].

The pathogenesis of stroke in COVID-19 infection is not clearly understood. Multiple mechanisms have been proposed including hypoxic injury, direct injury, immune injury, endothelial injury, embolism, DIC, necrotizing encephalopathy, vasculitis, and cardiomyopathy and activation of the coagulation pathway, with the latter being the more likely reason [7].

Activation of the coagulation pathway occurs early in the disease process and this leads to excessive production of proinflammatory cytokines believed to be responsible for multiorgan injuries. Imbalance of procoagulant-anticoagulant activity due to excessive inflammation is the proposed mechanism behind micro-thrombosis and DIC leading to thrombotic complications including pulmonary embolism and stroke. High D-dimer levels, often twice above the normal range, are noticed, reflecting the prothrombotic stage, and high D-dimer levels have been correlated with severe disease, worse prognosis, and higher mortality [8,9]. Studies have demonstrated that COVID-19 patients with stroke had high levels of D-dimer and C-reactive protein (CRP) than COVID-19 patients without stroke [10]. Our study included only patients with stroke and they all had high CRP and D-dimer at the time of the diagnosis of stroke. The acute inflammation caused by COVID-19 is probably prone to a hypercoagulable state that could lead to stroke.

In our series, multi-territorial infarctions were noted in all patients, suggesting arterial embolism, although large vessel occlusion has also been noted in patients with COVID-19 with or without traditional risk factors [11]. Hemorrhagic stroke is less common in COVID-19 [12]. Hence, one can debate about the pathogenesis of embolism vs. hypercoagulability regarding the cause of stroke in these patients. It seems likely that anticoagulation will play a substantial role in the management of stroke in COVID-19.

Angiotensin-converting enzyme 2 (ACE2) has been identified as a co-receptor for SARS-CoV-2 entry into cells, and it is expressed in many organs including the central nervous system (CNS). Neurotropism has been identified in coronaviruses causing SARS and the Middle East respiratory syndrome (MERS), and similarly, SARS-CoV-2 may invade the CNS through hematogenous spread or from peripheral nerve terminals and cause various neurologic manifestations [2,4]. Our patients were intubated and sedated, but as some studies have reported anosmia as a symptom of SARS-COV-2 infection, it supports the hypothesis of the invasion of the virus through the retrograde neuronal route.

The relationship between pneumonia and increased thrombosis is well established, but no clear mechanism has been established. In one study, critically ill H1N1 patients with acute respiratory distress syndrome (ARDS) were noted to have 17.9 times increased risk of venous thromboembolism (VTE) [13]. Elevated stroke risk in infections is likely due to the activation of clotting cascade by inflammation [14].

A comparison of our case series with other published studies regarding stroke in COVID-19 is presented in Table 2.

**Table 2:** Comparison of current series with other studies of stroke in COVID-19 patients CT: computed tomography; MRI: magnetic resonance imaging; COVID-19: coronavirus disease 2019

Study	Type of study	Diagnostic modality	Finding	Mean age of the study population (years)
Current study	Retrospective	CT	Acute cerebrovascular disease	61.5
Helms et al., 2020, France [4]	Prospective	MRI	Cerebral ischemic stroke	63
Klok et al., 2020, Netherlands [3]	Prospective	CT	Acute cerebrovascular disease	64
Li et al., 2020, China [10]	Retrospective	CT	Acute cerebrovascular disease	53.3
Lodigiani et al., 2020, Italy [15]	Retrospective	Report of treating physician	Acute cerebrovascular disease	66
Lu et al., 2020, China [16]	Retrospective	Not reported	Acute cerebrovascular disease	44
Mao et al., 2020, China [2]	Retrospective	CT	Acute cerebrovascular disease	52.7

All patients were intubated and sedated during the study; hence, consent was obtained from the HCP of all patients for the data collection and publication of this case report.

## Conclusions

Any patient with stroke or stroke-like symptoms should be screened for co-existing SARS-CoV-2 infection during this COVID-19 pandemic. Stroke patients are susceptible to severe COVID-19 infections and they usually have a worse prognosis. Critically ill patients with COVID-19 infection are at higher risk of developing stroke. Stroke should be considered in all patients presenting with altered mental status in the setting of COVID-19 infection. In patients with high D-dimer levels and those who also have traditional risk factors of stroke and acceptable bleeding risk, therapeutic anticoagulation may be considered.
